# Protective Effects of *Vitis coignetiae* Vine Stem Extract Against Carbon Tetrachloride-Induced Acute Liver Injury in Mice

**DOI:** 10.3390/antiox15050651

**Published:** 2026-05-21

**Authors:** Nam-Kyu Yoon, Jeongjun Lee, Hunsuk Chung, Jae-Kwang Kim, Sae-Kwang Ku

**Affiliations:** 1 Department of Anatomy and Histology, College of Korean Medicine, Daegu Haany University, Gyeongsan 38610, Republic of Korea; yoonnnamkyu@dhu.ac.kr; 2 GAPI BIO Co., Ltd., Hwaseong 18622, Republic of Korea; orglab@gapibio.co.kr (J.L.); hunsukchung@dongbangchem.co.kr (H.C.); 3 Department of Physiology, College of Korean Medicine, Daegu Haany University, Gyeongsan 38610, Republic of Korea

**Keywords:** *Vitis coignetiae* Pulliat ex Planch, carbon tetrachloride, acute liver injury, oxidative stress, inflammation

## Abstract

*Vitis coignetiae* Pulliat ex Planch, commonly referred to as “meoru” in Korea (crimson glory vine), is a grape species belonging to the Vitaceae family, native to East Asia. This study investigated the protective effects of a hot water extract prepared from the vine stems of *V. coignetiae* (CG) in a model of CCl_4_-induced acute liver injury. Mice received oral administration of CG (100, 200, and 400 mg/kg) or silymarin (200 mg/kg) once daily for 7 consecutive days, followed by intraperitoneal injection of CCl_4_ (0.5 mL/kg). CG attenuated CCl_4_-induced oxidative stress, as indicated by reduced hepatic malondialdehyde production and decreased 4-hydroxynonenal-positive cells. These effects were accompanied by restoration of antioxidant defense systems, including increased glutathione levels and superoxide dismutase and catalase activities, along with increased nuclear factor erythroid 2-related factor 2 (Nrf2) mRNA expression. Hepatic inflammatory responses were also attenuated by CG treatment, with reductions in TNF-α, interleukin (IL)-1β, and IL-6 levels, inflammatory cell infiltration, and nuclear factor-κB (NF-κB) mRNA expression. Furthermore, CG attenuated apoptotic cell death, as evidenced by decreased cleaved caspase-3-positive and cleaved poly(ADP-ribose) polymerase (PARP)-positive cells. CG also lowered serum aspartate aminotransferase, alanine aminotransferase, and γ-glutamyl transferase levels, and alleviated hepatocellular degeneration in histopathological analysis. Collectively, these findings suggest that CG may exert protective effects against CCl_4_-induced liver injury by regulating oxidative stress, inflammation, and apoptosis.

## 1. Introduction

The liver performs essential roles in metabolism, detoxification, and synthesis. However, these functions also make it vulnerable to toxic insults, as the metabolism of xenobiotics can generate reactive intermediates and reactive oxygen species (ROS), resulting in oxidative stress and hepatocellular injury [1]. This initial damage is further aggravated by inflammatory responses, in which both external and internal signals activate Kupffer cells and promote the release of pro-inflammatory cytokines [2]. If not properly controlled, these processes can exacerbate liver injury and contribute to further hepatic dysfunction. Therefore, alleviating early hepatocellular damage and inflammation is critical for preventing liver diseases.

Oxidative stress is a key mechanism underlying liver injury in various conditions, including drug-induced toxicity, chronic alcohol consumption, and fatty liver disease [1]. It promotes hepatocellular damage by inducing lipid peroxidation, depleting glutathione (GSH), and impairing antioxidant enzyme activities, thereby disrupting the liver’s defense systems. Nuclear factor erythroid 2-related factor 2 (Nrf2) is an important regulator of cellular antioxidant responses through interactions with antioxidant response elements (AREs). The Nrf2/ARE signaling pathway contributes to cellular antioxidant defense by increasing antioxidant enzyme expression and preserving GSH-mediated redox balance [3]. Based on this mechanism, natural products have attracted considerable attention as potential activators of the Nrf2 pathway, suggesting a promising strategy for protecting against oxidative liver injury [4].

In addition to oxidative stress, inflammatory responses are closely involved in the development of liver injury. Hepatic damage activates Kupffer cells and promotes the production of pro-inflammatory cytokines, including tumor necrosis factor-α (TNF-α) and interleukin (IL)-1, which contribute to the amplification of hepatic tissue injury [5]. This response promotes the recruitment of inflammatory cells, further aggravating liver damage. Moreover, ROS generated by Kupffer cells interacts with nitric oxide to form peroxynitrite, thereby exacerbating hepatocellular injury [5]. These inflammatory processes are closely associated with nuclear factor-κB (NF-κB), an important transcription factor involved in inflammatory gene expression and immune responses [6]. Activated NF-κB undergoes nuclear translocation and promotes inflammatory signaling, which contributes to the development and progression of liver injury.

Because of their antioxidant and anti-inflammatory activities, natural products have been extensively explored as potential candidates for the prevention and management of liver diseases [4,7]. *Vitis coignetiae* Pulliat ex Planch, commonly referred to as “meoru” in Korea (crimson glory vine), is a grapevine species in the Vitaceae family that is widely distributed throughout East Asia, including Korea [8,9]. Extracts derived from *V. coignetiae* contain diverse polyphenolic constituents, including anthocyanins and resveratrol, and are associated with multiple biological activities such as antioxidant, anti-inflammatory, and anticancer effects [8,9,10,11,12,13].

Because the fruits of *V. coignetiae* are commonly consumed, earlier studies have mainly investigated the biological properties of the fruit extracts and their major constituents [8,11]. However, despite their potential value, other underutilized parts, such as the vine stem, have received relatively little attention, and studies investigating their biological effects remain limited. Accordingly, the present study examined the hepatoprotective potential of CG extract in a CCl_4_-induced acute liver injury mouse model.

## 2. Materials and Methods

### 2.1. Chemicals and Preparations of CG Extract

CCl_4_ was supplied by Sigma-Aldrich (St. Louis, MO, USA; Cat. No. 270652; Batch No. 04049DE) and prepared at a ratio of 1:19 (*v*/*v*) in olive oil (Sigma-Aldrich; Cat. No. O1514; Lot. No. BCBW5235) prior to administration. Silymarin, used as a reference drug, was also obtained from Sigma-Aldrich (Cat. No. S0292; Lot. No. BCBF6608V) and suspended in distilled water (20 mg/mL) for oral administration.

CG extract was prepared through hot-water extraction. Briefly, the raw materials were subjected to two sequential hot water extractions, followed by filtration using a 1 μm filter (Filtertech, Daejeon, Republic of Korea; Cat. No. FT-WD-250-1). The resulting extract filtrate was then concentrated and spray-dried to produce a powdered extract with a final yield of 10%. For quality control, resveratrol was used as a marker compound, and its content in CG was determined to be 1.12 mg/g using high-performance liquid chromatography (HPLC) (Supplementary Information). Before oral administration, the dried extract was suspended in distilled water at concentrations of 10, 20, and 40 mg/mL.

### 2.2. Experimental Animals

Six-week-old male ICR mice were supplied by OrientBio (Seongnam, Republic of Korea). The animals were acclimated for 1 week before the experiment and maintained under controlled laboratory conditions (20–25 °C temperature; 30–35% humidity; 12 h light/dark cycle) with free access to standard laboratory chow (Purinafeed, Seongnam, Republic of Korea; Cat. No. 38057) and water. Animal experiments were reviewed and authorized by the Institutional Animal Care and Use Committee of Daegu Haany University (Approval No. DHU2024-026; 9 April 2024), and all procedures were conducted in compliance with institutional regulations for animal welfare and experimental ethics.

### 2.3. Experimental Design and Treatment

Following acclimation, the mice were randomly assigned to six experimental groups (*n* = 10/group) as follows: (1) intact vehicle control, (2) CCl_4_ control, (3) silymarin-treated group (200 mg/kg), (4) CG100 (100 mg/kg), (5) CG200 (200 mg/kg), and (6) CG400 (400 mg/kg). CG extract and silymarin were administered once daily by oral gavage for 7 consecutive days. During the same period, mice in the intact vehicle and CCl_4_ control groups received an equivalent volume of distilled water. One hour after the final administration, acute liver injury was induced by intraperitoneal injection of CCl_4_ diluted in olive oil (1:19, *v*/*v*) into the lower right abdominal cavity. The injection volume was 10 mL/kg, corresponding to 0.5 mL/kg of pure CCl_4_. Mice in the intact vehicle control group received the same volume of olive oil via intraperitoneal injection. After 24 h of CCl_4_ administration, all animals were anesthetized with 2–3% isoflurane (Hana Pharm Co., Hwaseong, Republic of Korea) delivered through an inhalation anesthesia system (Surgivet, Waukesha, WI, USA) connected to a rodent ventilator (Model 687, Harvard Apparatus, Cambridge, UK), followed by euthanasia.

### 2.4. Measurements of Body and Liver Weight

Body weights were recorded daily during the experimental period, starting one day before the first administration of test substances and continuing until 24 h after CCl_4_ injection. Following euthanasia, the liver was removed after gross examination and weighed. Relative liver weight was calculated as the percentage of liver weight relative to body weight at the time of sacrifice.

### 2.5. Serum Biochemistry

At the time of sacrifice, blood samples were collected, and serum was separated by centrifugation at 12,500 rpm for 10 min using clot activator tubes. Serum aspartate aminotransferase (AST), alanine aminotransferase (ALT), and γ-glutamyl transferase (γ-GTP) levels were analyzed with an automated biochemical analyzer (Dri-Chem NX500i, Fujifilm Corporation, Tokyo, Japan) following the manufacturer’s recommended protocol. The measured values were presented in IU/L.

### 2.6. Measurements of Hepatic Inflammatory Cytokines

Liver tissues were homogenized in pre-cooled radioimmunoprecipitation assay buffer and then maintained on ice for 30 min. Tissue lysates were subjected to two rounds of centrifugation at 20,000× *g* for 15 min at 4 °C, after which the clarified supernatants were collected.

Hepatic TNF-α (Cat. No. MBS825075), IL-1β (Cat. No. MBS824958), and IL-6 (Cat. No. MBS730957) concentrations were quantified using commercially available enzyme-linked immunosorbent assay (ELISA) kits obtained from MyBioSource (San Diego, CA, USA) following the supplier’s instructions. The final values were reported in ng/L.

### 2.7. Quantitative Real-Time PCR Analysis

Total RNA from liver tissues was isolated using TRIzol reagent (Invitrogen, Carlsbad, CA, USA; Cat. No. 15596026). The extracted RNA was reverse-transcribed into cDNA using a High-Capacity cDNA Reverse Transcription Kit (Thermo Fisher Scientific, Rockford, IL, USA; Cat. No. 4368813). Quantitative real-time PCR was subsequently carried out using a CFX96^TM^ Real-Time PCR System (Bio-Rad, Hercules, CA, USA). Primer sets used for qPCR analysis were purchased from OriGene Technologies (Rockville, MD, USA), and detailed primer information is provided in Table 1. Expression levels of Nrf2, NF-κB, TNF-α, IL-1β, and IL-6 were analyzed relative to β-actin, which served as the reference gene. Relative gene expression was determined by the 2^−ΔΔCt^ method [14].

### 2.8. Measurement of Lipid Peroxidation

To evaluate hepatic lipid peroxidation, malondialdehyde (MDA) levels were measured using a thiobarbituric acid-reactive substances (TBARS) assay according to a previously described procedure [15]. Liver tissues were homogenized in ice-cold Tris-HCl buffer (0.01 M, pH 7.4) and centrifuged at 12,000× *g* for 15 min. After centrifugation, the separated supernatants were preserved at −150 °C prior to biochemical analysis. Following the TBARS assay procedure, the resulting MDA–TBA adduct was analyzed by measuring the absorbance of the reaction mixture at 525 nm using a UV/Vis spectrophotometer (Optizen POP, Mecasys, Daejeon, Republic of Korea). MDA levels were normalized to protein content and expressed as nM/mg protein.

### 2.9. Measurement of Antioxidant Defense Systems

Liver homogenates were treated with 0.1 mL of 25% trichloroacetic acid (Merck, West Point, PA, USA) followed by centrifugation at 4200 rpm for 40 min at 4 °C. Hepatic GSH content was determined spectrophotometrically at 412 nm using Ellman’s reagent containing 5,5′-dithiobis-(2-nitrobenzoic acid) (DTNB; Sigma-Aldrich; Cat. No. D8130). Superoxide dismutase (SOD) activity was evaluated using a xanthine-xanthine oxidase system, in which superoxide radicals reduce nitroblue tetrazolium (NBT) to formazan. The inhibitory effect on NBT reduction was monitored spectrophotometrically at 560 nm, and SOD activity was expressed as U/mg protein. One unit of SOD activity corresponded to the level of enzyme activity producing 50% inhibition of NBT reduction within 1 min. Catalase (CAT) activity was assessed by measuring the decomposition of H_2_O_2_ at 240 nm with a spectrophotometer. One unit of CAT activity was defined as the enzyme activity resulting in the decomposition of 1 μmol of H_2_O_2_ per minute under pH 7.8 and 25 °C conditions, and the activity was expressed as U/mg protein.

### 2.10. Histopathological Analysis

Liver tissues obtained from the left lateral lobe were immersed in 10% neutral buffered formalin for fixation and subsequently processed for paraffin embedding after 24 h. Paraffin blocks were serially sectioned into 3–4 μm slices and stained with hematoxylin and eosin (H&E). Histopathological observations were performed using an Eclipse 80i microscope (Nikon, Tokyo, Japan), and digital image analysis was conducted with the *i*Solution FL software package (ver. 9.1; IMT *i*Solution Inc., Burnaby, BC, Canada). All histological evaluations were performed in a blinded manner. Severity of liver injury was assessed according to the modified histological activity index (HAI) scoring system, including necrotic, apoptotic, and inflammatory alterations [15,16]. Quantitative analysis was also performed to determine the extent of degenerative hepatic parenchyma, the number of degenerative hepatocytes, and infiltrating inflammatory cells, and the results were expressed as %/mm^2^, cells/1000 hepatocytes, and cells/mm^2^, respectively.

### 2.11. Immunohistochemistry

Immunohistochemical staining was conducted by the avidin–biotin complex (ABC) method. For antigen retrieval, tissue sections were heated in 10 mM citrate buffer (pH 6.0) at 95–100 °C. Endogenous peroxidase activity was quenched by treatment with 0.3% H_2_O_2_ in methanol for 30 min, followed by blocking with normal horse serum to minimize non-specific binding. Tissue sections were incubated overnight at 4 °C with primary antibodies against cleaved caspase-3 (1:400, Cell Signaling Technology, Danvers, MA, USA; Cat. No. 9661), cleaved poly(ADP-ribose) polymerase (PARP; 1:100; Santa Cruz Biotechnology Inc., Santa Cruz, CA, USA; Cat. No. sc-23461), nitrotyrosine (NT; 1:200; Millipore, Temecula, CA, USA; Cat. No. 06-284), and 4-hydroxy-2-nonenal (4-HNE; 1:100; Abcam, Cambridge, UK; Cat. No. ab46545). After incubation with biotinylated secondary antibodies and ABC reagents (Vectastain Elite ABC Kit, Cat. No. PK-6200, Vector Laboratories, Burlingame, CA, USA), a peroxidase substrate kit (Cat. No. SK-4100, Vector Laboratories) was applied for chromogenic detection. Immunopositive hepatocytes were defined as cells exhibiting immunoreactivity in more than 20% of the cell area and were quantified as cells per 1000 hepatocytes using an automated image analysis system. All immunohistochemical evaluations were conducted under blinded conditions.

### 2.12. Statistical Analysis

Results are expressed as mean ± standard deviation (SD) for each group (*n* = 10). Statistical analyses were carried out using SPSS for Windows (Version 18.0; IBM Corp., Armonk, NY, USA). Homogeneity of variance was evaluated with Levene’s test prior to group comparisons. When equal variance assumptions were met, one-way analysis of variance (ANOVA) was used to evaluate the statistical differences among groups, and Tukey’s honestly significant difference (HSD) was subsequently applied for post hoc analysis. If variance homogeneity was not satisfied, Dunnett’s T3 test was applied. Statistical significance was defined at *p* < 0.05.

## 3. Results

### 3.1. Effects of CG on Body and Liver Weights

**Figure 1:**
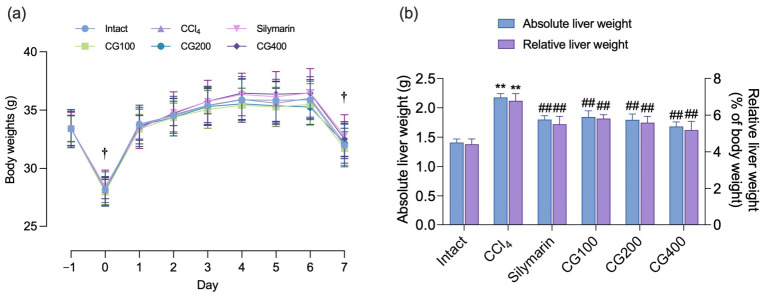
Effects of vine stem extract of *Vitis coignetiae* (CG) on body and liver weights. (**a**) Body weights were measured daily from 1 day before the first administration to 24 h after carbon tetrachloride (CCl_4_) injection. (**b**) Absolute (g) and relative liver weight (% of body weight) were measured at sacrifice. Values are presented as mean ± standard deviation (SD) (*n* = 10). ^†^ All animals were fasted overnight. ** *p* < 0.01 vs. intact vehicle control; ^##^ *p* < 0.01 vs. CCl_4_ control.

Body weights were monitored daily beginning 1 day before treatment initiation and continuing until the end of the experiment (Figure 1a). Over the 7-day experimental period, body weight did not differ significantly between the intact vehicle and CCl_4_ control groups. Similarly, administration of silymarin or CG (100–400 mg/kg) did not produce significant alterations in body weight compared with the CCl_4_ control group. At the time of sacrifice, absolute and relative liver weights were evaluated. Mice exposed to CCl_4_ showed a significant increase in absolute and relative liver weight compared with the intact vehicle group, whereas treatment with silymarin or CG (100–400 mg/kg) significantly attenuated this increase (Figure 1b).

### 3.2. Effects of CG on Serum AST, ALT, and γ-GTP Levels

**Figure 2:**
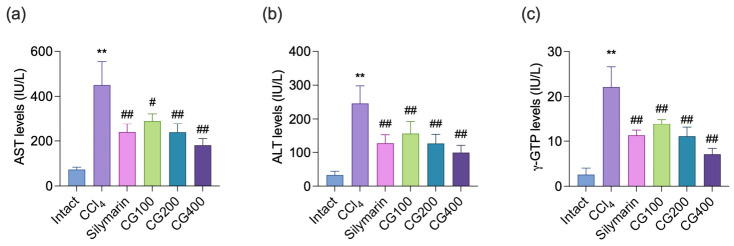
Effects of CG on serum biochemical parameters. Serum levels of (**a**) aspartate aminotransferase (AST), (**b**) alanine aminotransferase (ALT), and (**c**) γ-glutamyl transferase (γ-GTP) were measured using an automated biochemical analyzer. Values are presented as mean ± SD (*n* = 10). ** *p* < 0.01 vs. intact vehicle control; ^#^ *p* < 0.05, ^##^ *p* < 0.01 vs. CCl_4_ control.

Serum AST, ALT, and γ-GTP levels were markedly elevated following CCl_4_ administration compared with those in the intact vehicle group (Figure 2). Treatment with CG (100–400 mg/kg) significantly attenuated these increases in a dose-dependent manner relative to the CCl_4_ control group. In addition, the hepatoprotective effects observed in the CG (100–400 mg/kg)-treated groups were comparable to those of the silymarin-treated group.

### 3.3. Effects of CG on Hepatic Expressions of Inflammatory Cytokines

**Figure 3:**
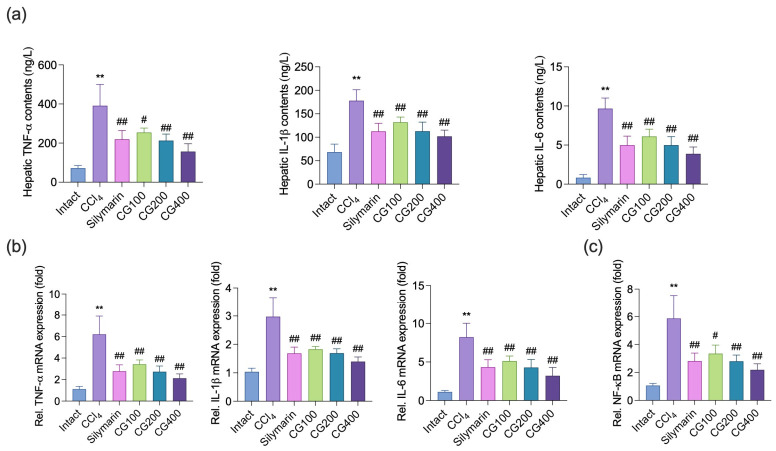
Effects of CG on hepatic inflammatory responses. (**a**) Hepatic tumor necrosis factor-α (TNF-α), interleukin (IL)-1β, and IL-6 levels were quantified by commercial enzyme-linked immunosorbent assay (ELISA) kits. (**b**,**c**) qRT-PCR analysis was performed to evaluate the relative mRNA expression of TNF-α, IL-1β, IL-6, and nuclear factor-κB (NF-κB). Values are presented as mean ± SD (*n* = 10). ** *p* < 0.01 vs. intact vehicle control; ^#^ *p* < 0.05, ^##^ *p* < 0.01 vs. CCl_4_ control.

Compared with the intact vehicle group, CCl_4_ administration markedly elevated hepatic TNF-α, IL-1β, and IL-6 levels at both the protein and mRNA levels in hepatic tissue (Figure 3a,b). However, compared with the CCl_4_ control group, CG treatment (100–400 mg/kg) significantly attenuated inflammatory cytokine production and corresponding mRNA expression levels in a dose-dependent manner. Notably, the effects observed in all CG-treated groups were comparable to those in the silymarin-treated group. In addition, mRNA expression of NF-κB, a transcription factor associated with pro-inflammatory cytokine regulation, was significantly increased following CCl_4_ treatment. In contrast, CG treatment (100–400 mg/kg) markedly suppressed its expression in a dose-dependent manner (Figure 3c).

### 3.4. Effects of CG on Hepatic Lipid Peroxidation and Antioxidant Defense Systems

**Figure 4:**
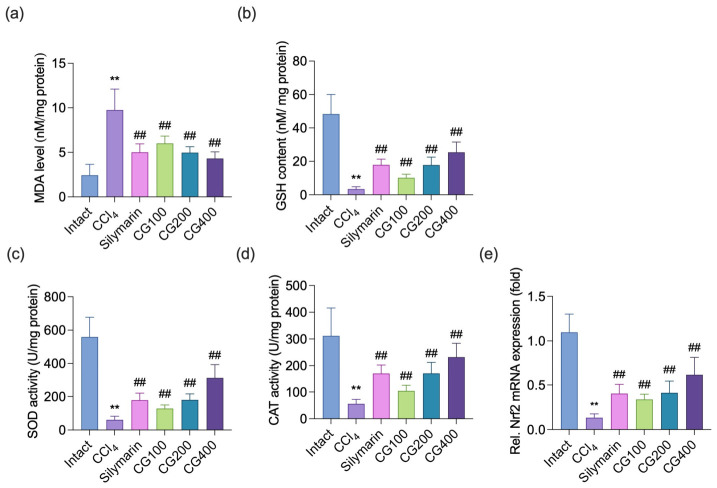
Effects of CG on oxidative stress and antioxidant defense systems. (**a**) Hepatic malondialdehyde (MDA) levels were evaluated using a thiobarbituric acid reactive substances (TBARS) assay. Hepatic glutathione (GSH) content (**b**), superoxide dismutase (SOD) activity (**c**), and catalase (CAT) activity (**d**) were determined spectrophotometrically. (**e**) Relative nuclear factor erythroid 2–related factor 2 (Nrf2) mRNA expression was analyzed by qRT-PCR. Values are presented as mean ± SD (*n* = 10). ** *p* < 0.01 vs. intact vehicle control; ^##^ *p* < 0.01 vs. CCl_4_ control.

Compared with the intact vehicle group, CCl_4_ administration markedly exacerbated hepatic lipid peroxidation, evidenced by elevated MDA levels (Figure 4a). This was accompanied by a significant reduction in GSH content and antioxidant enzyme activities, including SOD and CAT (Figure 4b–d). CG treatment (100–400 mg/kg) significantly attenuated these alterations, reducing lipid peroxidation while significantly restoring GSH levels together with SOD and CAT activities in a dose-dependent manner. Consistently, Nrf2 mRNA expression, a key transcription factor regulating antioxidant genes, was significantly downregulated by CCl_4_ treatment, whereas it was significantly increased by CG administration (100–400 mg/kg) (Figure 4e).

### 3.5. Effects of CG on Hepatic Histopathological Alterations, Apoptosis, and Oxidative Damage

**Figure 5:**
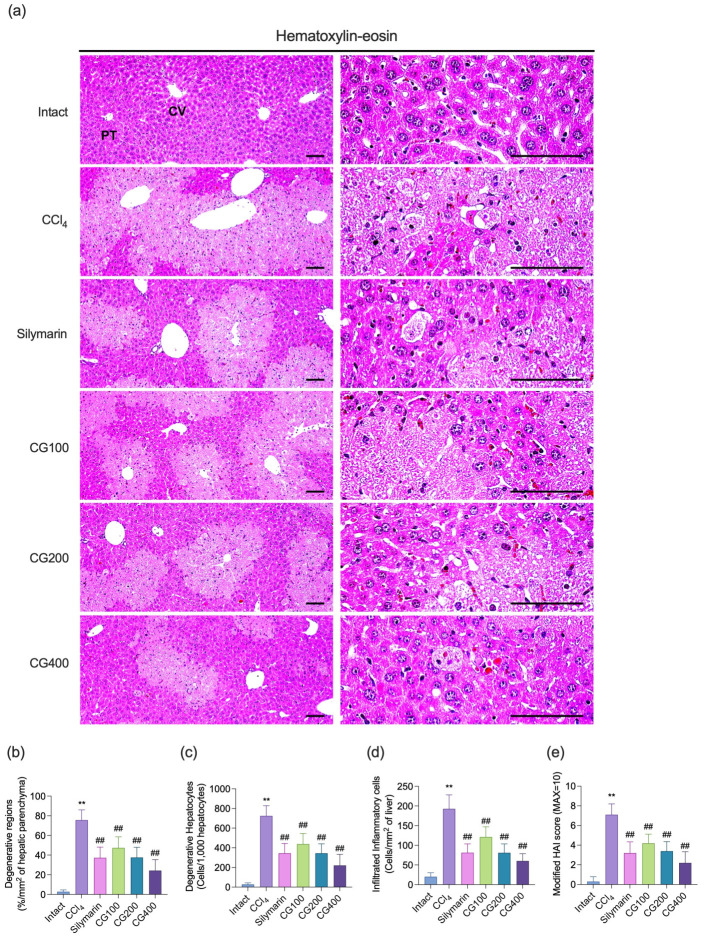
Effects of CG on histopathological profiles of liver tissues. (**a**) Representative images of hematoxylin and eosin (H&E)-stained liver tissues. Scale bars = 200 μm. Histopathological changes were evaluated by measuring (**b**) degenerative regions, (**c**) degenerative hepatocytes, (**d**) infiltrated inflammatory cells, and (**e**) modified histological activity index (HAI) score. Values are presented as mean ± SD (*n* = 10). ** *p* < 0.01 vs. intact vehicle control; ^##^ *p* < 0.01 vs. CCl_4_ control. CV, central vein; PT, portal triad.

**Figure 6:**
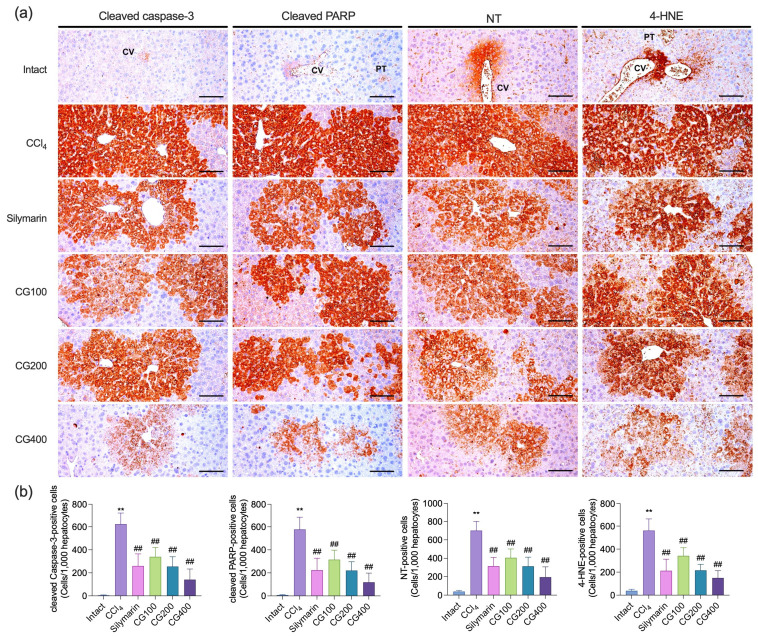
Effects of CG on immunohistochemical profiles of liver tissues. (**a**) Representative images of immunohistochemistry. Cleaved caspase-3 and cleaved poly(ADP-ribose) polymerase (PARP) staining were used to evaluate apoptotic cell death, whereas nitrotyrosine (NT) and 4-hydroxynonenal (4-HNE) staining were used as indicators of oxidative and nitrative stress. Scale bars = 200 μm. (**b**) Positive cells for cleaved caspase-3, cleaved PARP, NT, and 4-HNE were quantitatively analyzed using an automated image analysis system. Values are presented as mean ± SD (*n* = 10). CV, central vein; PT, portal triad. ** *p* < 0.01 vs. intact vehicle control; ^##^ *p* < 0.01 vs. CCl_4_ control.

CCl_4_ treatment markedly induced hepatic histopathological alterations, including hepatocellular vacuolation, lipid droplet accumulation, centrilobular necrosis, and inflammatory cell infiltration, compared to the intact vehicle control group (Figure 5a). These alterations were also accompanied by significant increases in degenerative regions, degenerative hepatocyte counts, inflammatory cell infiltration, and modified HAI score (Figure 5b–e). In addition, CCl_4_ treatment significantly increased apoptotic cell death, as evidenced by elevated numbers of cleaved caspase-3- and cleaved PARP-positive cells, along with increased oxidative damage, indicated by higher numbers of NT- and 4-HNE-positive cells (Figure 6). CG treatment (100–400 mg/kg) significantly attenuated these alterations, reducing histopathological damage, apoptotic cell death, and oxidative damage in a dose-dependent manner. Notably, all CG-treated groups exhibited effects comparable to those of the silymarin-treated group.

## 4. Discussion

CCl_4_ is a well-established hepatotoxic agent. After administration, CCl_4_ is metabolized by cytochrome P450 enzymes, predominantly by CYP2E1, to generate highly reactive trichloromethyl radicals, which are further converted to trichloromethyl peroxyl radicals in the presence of oxygen [17]. These reactive intermediates initiate oxidative stress by triggering lipid peroxidation, which disrupts membrane integrity, impairs mitochondrial function, and depletes endogenous antioxidant defenses [18]. Oxidative hepatic injury is also associated with inflammatory signaling activation, including NF-κB, which promotes the production of pro-inflammatory cytokines, including TNF-α, IL-1β, and IL-6. Persistent oxidative and inflammatory responses further contribute to hepatocyte death [19]. Based on these characteristics, the CCl_4_-induced acute liver injury model offers several advantages, including the rapid induction of liver injury within 24 h after administration, high reproducibility, and cost-effectiveness. In addition, this experimental model reproduces various functional, metabolic, and histopathological changes associated with acute liver injury [19]. Moreover, repeated administration over 8–12 weeks is also used as a classical model of chronic liver disease [19,20]. Therefore, the CCl_4_ model is considered a reliable and widely accepted tool for investigating liver injury. In the present study, acute liver injury was induced in mice through intraperitoneal administration of CCl_4_ (0.5 mL/kg) in mice, as previously described [15,21], and this model was used to assess the hepatoprotective potentials of CG. Silymarin, a bioactive flavonolignan mixture derived from milk thistle plant (*Silybum marianum*), is well known for its hepatoprotective effects, primarily attributed to its antioxidant properties, and it has demonstrated hepatoprotective effects against CCl_4_-induced acute liver injury [22,23]. For this reason, silymarin has been widely used as a positive control in animal studies at doses of 100–200 mg/kg. Accordingly, silymarin was administered at 200 mg/kg in the present study [15,24,25,26].

Lipid peroxidation is initiated by excessive ROS generated during CCl_4_ metabolism, and involves a chain reaction of membrane polyunsaturated fatty acid oxidation, leading to membrane damage and the accumulation of reactive lipid intermediates [17]. This process impairs mitochondrial function and disrupts endoplasmic reticulum homeostasis while generating toxic aldehydes such as MDA and 4-HNE. Although MDA is commonly utilized as a marker of lipid peroxidation, 4-HNE acts as a key effector of hepatocellular damage by covalently modifying proteins, inhibiting mitochondrial respiration and endoplasmic reticulum enzymes, and disrupting calcium homeostasis, thereby amplifying oxidative liver injury [27]. In the present study, hepatic MDA levels and immunoreactivity of 4-HNE were markedly elevated following CCl_4_ administration, reflecting enhanced lipid peroxidation (Figure 4a and Figure 6). However, CG treatment markedly attenuated these changes, suggesting that the hepatoprotective effects of CG are closely related to reduced lipid peroxidation and improved redox balance. Resveratrol detected in CG may represent one of several constituents potentially contributing to these effects (Figure S1) [13]. Previous studies have shown that resveratrol reduces MDA accumulation in thioacetamide- and CCl_4_-induced liver injury models, suggesting that resveratrol present in CG may partially contribute to the reduction of lipid peroxidation [28,29].

The decrease in antioxidant enzyme activities is attributed to multiple mechanisms, including excessive ROS generation, oxidative modification of proteins, and inactivation of enzymes by lipid peroxidation-derived reactive aldehydes such as 4-HNE [1,30]. Consistently, reductions in these antioxidant defenses have been widely reported in CCl_4_-induced liver injury models [15,21,25,31]. Consistent with these observations, CCl_4_ exposure in the present study markedly reduced hepatic GSH levels together with SOD and CAT activities, reflecting impairment of the antioxidant defense system (Figure 4). Notably, CG treatment markedly restored these parameters, suggesting that the hepatoprotective effects of CG may be related to improved antioxidant defense and preservation of redox homeostasis.

Because Nrf2 is involved in oxidative stress, inflammation, and fibrosis, it has been regarded as a potential therapeutic target in various liver diseases [3,32]. Under oxidative stress conditions, Nrf2 translocates into the nucleus and promotes ARE-dependent antioxidant gene expression, thereby contributing to the restoration of redox homeostasis and protection against cellular damage [32]. Consistently, activation of the Nrf2/ARE signaling pathway has been widely reported to exert hepatoprotective effects against CCl_4_-induced liver injury by enhancing antioxidant defense systems, suppressing oxidative stress, and attenuating inflammatory and apoptotic responses [25,31,33]. In contrast, hepatocyte-specific Nrf2 deficiency has been reported to aggravate CCl_4_-induced oxidative injury and inflammatory responses, contributing to the progression of liver damage and fibrosis [34,35]. In the present study, CG administration significantly upregulated Nrf2 mRNA expression compared to the CCl_4_ control group (Figure 4e). Although Nrf2 activity is generally evaluated based on protein expression or transcriptional regulation, several studies have reported that increased Nrf2 mRNA expression is linked to hepatoprotective responses in experimental hepatic injury induced by CCl_4_. In these models, Nrf2 mRNA expression is suppressed under oxidative stress conditions, but restored by protective interventions, correlating with enhanced antioxidant capacity and reduced liver damage, often accompanied by increased expression of antioxidant-related genes, including heme oxygenase-1 (HO-1) and NAD(P)H-quinone oxidoreductase 1 (NQO1) [25,26,36]. Therefore, the restoration of Nrf2 mRNA expression by CG following CCl_4_ exposure may be associated with enhanced hepatic antioxidant defense responses. However, because the chemical profile of *V. coignetiae* vine stems has not been sufficiently characterized, the specific constituents responsible for Nrf2 activation remain unclear. Although resveratrol identified in CG has been reported to activate Nrf2 signaling in various experimental models [13,37], further mechanistic and phytochemical analyses are needed to better understand the active constituents and their involvement in Nrf2-associated pathways.

Inflammation is a key driver of liver injury progression in CCl_4_-induced hepatotoxicity. Reactive intermediates generated during CCl_4_ metabolism initiate oxidative damage, which subsequently activates Kupffer cells and promotes the production of pro-inflammatory cytokines, particularly TNF-α, IL-1β, and IL-6 [38]. These events not only reflect hepatic inflammation but also facilitate infiltration of immune cells, including neutrophils and monocytes, thereby aggravating tissue injury [39,40]. Consistently, CCl_4_ administration increased hepatic cytokine levels and inflammatory cell infiltration, whereas CG treatment attenuated these changes (Figure 3 and Figure 5). In addition, CG reduced NT immunoreactivity, suggesting attenuation of peroxynitrite-mediated nitrosative stress, thereby limiting oxidative and inflammation-driven liver damage (Figure 6). Furthermore, NF-κB, a key transcription factor involved in inflammatory responses and cytokine regulation, showed markedly increased mRNA expression following CCl_4_ administration, whereas CG treatment reduced its expression (Figure 3c), suggesting possible involvement in the modulation of inflammatory signaling pathways. Although anthocyanins derived from *V. coignetiae* fruits have been reported to inhibit NF-κB signaling [8,11], and both leaf extracts and resveratrol have demonstrated anti-inflammatory effects in liver disease models via NF-κB inhibition [12,20,29,41], these lines of evidence remain insufficient to explain the effects of vine stem extracts due to the lack of direct studies on this plant part. Accordingly, additional phytochemical and mechanistic investigations are needed to clarify the active constituents of CG and to further characterize its molecular actions at the protein level, including NF-κB phosphorylation, nuclear translocation, and DNA-binding activity.

Following oxidative stress and inflammatory responses, progressive hepatocellular degeneration and cell death are major pathological features of acute liver injury induced by CCl_4_. In the present study, these alterations were evidenced by significant increases in degenerative region area, the number of degenerative hepatocytes, and HAI scores, along with marked elevations in serum AST, ALT, and γ-GTP levels (Figure 2 and Figure 5). These changes are associated with mitochondrial dysfunction and inflammatory signaling, particularly TNF-α, which can promote apoptotic pathways in injured hepatocytes [17]. Cleaved caspase-3 and cleaved PARP are widely recognized indicators of apoptotic cell death. In this study, CCl_4_ administration markedly increased the number of cells immunoreactive for cleaved caspase-3 and cleaved PARP, suggesting enhanced hepatocellular apoptosis. Importantly, CG treatment attenuated the elevation of serum liver injury markers and improved histopathological alterations, including reductions in degenerative regions, degenerative hepatocytes, and HAI scores (Figure 2 and Figure 5). Furthermore, CG reduced cleaved caspase-3 and cleaved PARP immunoreactivity, indicating attenuation of apoptotic cell death (Figure 6). Taken together, these findings suggest that CG exerts hepatoprotective effects by attenuating oxidative stress and inflammatory responses, thereby reducing apoptosis and overall hepatocellular damage.

Despite the protective effects of CG against liver injury observed in the present study, several limitations should be considered. Although resveratrol was identified in CG and may partially contribute to its biological activities, the active constituents responsible for the hepatoprotective effects of CG remain unclear. Therefore, further phytochemical and mechanistic studies using individual compounds are required. In addition, because only the mRNA expression levels of Nrf2 and NF-κB were evaluated in the present study, the precise regulation of these signaling pathways could not be fully confirmed at the protein level. Further studies investigating phosphorylation, nuclear translocation, and DNA-binding activity are needed to clarify the underlying molecular mechanisms of CG. Moreover, although no mortality or apparent adverse effects were observed under the present experimental conditions, the absence of a CG-alone group in normal mice limits the evaluation of its basal effects and potential hepatotoxicity, indicating the need for additional repeated-dose toxicity studies. Finally, because the present study examined the hepatoprotective effects of CG only at an early time point (24 h after CCl_4_ administration), further pharmacokinetic and extended experimental studies are required to clarify the clinically relevant dose range and persistence of the hepatoprotective effects of CG.

## 5. Conclusions

In conclusion, CG exerted significant hepatoprotective effects against CCl_4_-induced acute liver injury, as evidenced by improvements in serum biochemical markers and histopathological alterations. These protective effects may involve reduced oxidative stress and inflammatory responses, along with suppression of hepatocellular apoptosis. Collectively, the present results indicate that CG may serve as a potential preventive strategy against acute liver injury.

## Figures and Tables

**Table 1:** Information on primer sequences used in RT-qPCR.

Target (Cat. No.)	Direction	Primer Sequences (5′⟶3′)	GenBank Accession Number
Nrf2 (MP209070)	Forward Reverse	CAGCATAGAGCAGGACATGGAG, GAACAGCGGTAGTATCAGCCAG	NM_010902
NF-κB (MP209060)	Forward Reverse	GCTGCCAAAGAAGGACACGACA, GGCAGGCTATTGCTCATCACAG	NM_008689
TNF-α (MP217748)	Forward Reverse	GGTGCCTATGTCTCAGCCTCTT, GCCATAGAACTGATGAGAGGGAG	NM_013693
IL-1β (MP206724)	Forward Reverse	TGGACCTTCCAGGATGAGGACA, GTTCATCTCGGAGCCTGTAGTG	NM_008361
IL-6 (MP206798)	Forward Reverse	TACCACTTCACAAGTCGGAGGC, CTGCAAGTGCATCATCGTTGTTC	NM_031168
β-actin (MP200232)	Forward Reverse	CATTGCTGACAGGATGCAGAAGG, TGCTGGAAGGTGGACAGTGAGG	NM_007393

Nrf2, nuclear factor erythroid 2-related factor 2; NF-κB, nuclear factor-κB; TNF-α, tumor necrosis factor-α; IL, interleukin.

## Data Availability

All data supporting the findings of this study are included in the article and its Supplementary Materials.

## Supplementary Materials

The following supporting information can be downloaded at: https://www.mdpi.com/article/10.3390/antiox15050651/s1, Figure S1: Identification of resveratrol in CG extract using high-performance liquid chromatography (HPLC) analysis. (a) Chromatogram of resveratrol standard. (b) Chromatogram of CG.

## References

[1] Cichoz-Lach H., Michalak A. (2014). Oxidative stress as a crucial factor in liver diseases. World J. Gastroenterol..

[2] Roberts R.A., Ganey P.E., Ju C., Kamendulis L.M., Rusyn I., Klaunig J.E. (2007). Role of the Kupffer cell in mediating hepatic toxicity and carcinogenesis. Toxicol. Sci..

[3] Shin S.M., Yang J.H., Ki S.H. (2013). Role of the Nrf2-ARE pathway in liver diseases. Oxidative Med. Cell. Longev..

[4] Jadeja R.N., Upadhyay K.K., Devkar R.V., Khurana S. (2016). Naturally Occurring Nrf2 Activators: Potential in Treatment of Liver Injury. Oxidative Med. Cell. Longev..

[5] Kolios G., Valatas V., Kouroumalis E. (2006). Role of Kupffer cells in the pathogenesis of liver disease. World J. Gastroenterol..

[6] Liu T., Zhang L., Joo D., Sun S.C. (2017). NF-κB signaling in inflammation. Signal Transduct. Target. Ther..

[7] Yan J., Nie Y., Luo M., Chen Z., He B. (2021). Natural Compounds: A Potential Treatment for Alcoholic Liver Disease?. Front. Pharmacol..

[8] Lu J.N., Lee W.S., Yun J.W., Kim M.J., Kim H.J., Kim D.C., Jeong J.H., Choi Y.H., Kim G.S., Ryu C.H. (2013). Anthocyanins from *Vitis coignetiae* Pulliat Inhibit Cancer Invasion and Epithelial-Mesenchymal Transition, but These Effects Can Be Attenuated by Tumor Necrosis Factor in Human Uterine Cervical Cancer HeLa Cells. Evid. Based Complement. Altern. Med..

[9] Takayama F., Nakamoto K., Kawasaki H., Mankura M., Egashira T., Ueki K., Hasegawa A., Okada S., Mori A. (2009). Beneficial effects of *Vitis coignetiae* Pulliat leaves on nonalcoholic steatohepatitis in a rat model. Acta Med. Okayama.

[10] Jeong H.-J., Park S.-B., Kim S.-A., Kim H.-K. (2007). Total Polyphenol Content and Antioxidative Activity of Wild Grape (*Vitis coignetiae*) Extracts Depending on Ethanol Concentrations. J. Korean Soc. Food Sci. Nutr..

[11] Paramanantham A., Kim M.J., Jung E.J., Kim H.J., Chang S.H., Jung J.M., Hong S.C., Shin S.C., Kim G.S., Lee W.S. (2020). Anthocyanins Isolated from *Vitis coignetiae* Pulliat Enhances Cisplatin Sensitivity in MCF-7 Human Breast Cancer Cells through Inhibition of Akt and NF-κB Activation. Molecules.

[12] Pak W., Takayama F., Hasegawa A., Mankura M., Egashira T., Ueki K., Nakamoto K., Kawasaki H., Mori A. (2012). Water extract of *Vitis coignetiae* Pulliat leaves attenuates oxidative stress and inflammation in progressive NASH rats. Acta Med. Okayama.

[13] Kim S.J., Lee Y.H., Han M.D., Mar W., Kim W.K., Nam K.W. (2010). Resveratrol, purified from the stem of *Vitis coignetiae* Pulliat, inhibits food intake in C57BL/6J Mice. Arch. Pharmacal Res..

[14] Livak K.J., Schmittgen T.D. (2001). Analysis of relative gene expression data using real-time quantitative PCR and the 2^−ΔΔCT^ Method. Methods.

[15] Kim J.S., Jegal K.H., Park H.R., Choi B.R., Kim J.K., Ku S.K. (2023). A Mixture of Fermented Schizandrae Fructus Pomace and Hoveniae Semen cum Fructus Extracts Synergistically Protects against Oxidative Stress-Mediated Liver Injury. Antioxidants.

[16] Ishak K., Baptista A., Bianchi L., Callea F., De Groote J., Gudat F., Denk H., Desmet V., Korb G., MacSween R.N. (1995). Histological grading and staging of chronic hepatitis. J. Hepatol..

[17] Weber L.W., Boll M., Stampfl A. (2003). Hepatotoxicity and mechanism of action of haloalkanes: Carbon tetrachloride as a toxicological model. Crit. Rev. Toxicol..

[18] Basu S. (2003). Carbon tetrachloride-induced lipid peroxidation: Eicosanoid formation and their regulation by antioxidant nutrients. Toxicology.

[19] Mao J., Tan L., Tian C., Wang W., Zhang H., Zhu Z., Li Y. (2024). Research progress on rodent models and its mechanisms of liver injury. Life Sci..

[20] Chavez E., Reyes-Gordillo K., Segovia J., Shibayama M., Tsutsumi V., Vergara P., Moreno M.G., Muriel P. (2008). Resveratrol prevents fibrosis, NF-κB activation and TGF-β increases induced by chronic CCl_4_ treatment in rats. J. Appl. Toxicol..

[21] Lee Y.S., Cho I.J., Kim J.W., Lee M.K., Ku S.K., Choi J.S., Lee H.J. (2019). Hepatoprotective effects of blue honeysuckle on CCl_4_-induced acute liver damaged mice. Food Sci. Nutr..

[22] Muriel P., Mourelle M. (1990). Prevention by silymarin of membrane alterations in acute CCl_4_ liver damage. J. Appl. Toxicol..

[23] Vargas-Mendoza N., Madrigal-Santillan E., Morales-Gonzalez A., Esquivel-Soto J., Esquivel-Chirino C., Garcia-Luna Y.G.-R.M., Gayosso-de-Lucio J.A., Morales-Gonzalez J.A. (2014). Hepatoprotective effect of silymarin. World J. Hepatol..

[24] Hsu Y.W., Tsai C.F., Chuang W.C., Chen W.K., Ho Y.C., Lu F.J. (2010). Protective effects of silica hydride against carbon tetrachloride-induced hepatotoxicity in mice. Food Chem. Toxicol..

[25] Iqbal N., Zubair H.M., Almutairi M.H., Abbas M., Akhtar M.F., Aleya L., Kamel M., Saleem A., Jabeen Q., Noreen S. (2022). Hepatoprotective effect of *Cordia rothii* extract against CCl_4_-induced oxidative stress via Nrf2-NFκB pathways. Biomed. Pharmacother..

[26] Peng C., Zhou Z.M., Li J., Luo Y., Zhou Y.S., Ke X.H., Huang K.E. (2019). CCl_4_-Induced Liver Injury Was Ameliorated by Qi-Ge Decoction through the Antioxidant Pathway. Evid. Based Complement. Altern. Med..

[27] Poli G., Albano E., Dianzani M.U. (1987). The role of lipid peroxidation in liver damage. Chem. Phys. Lipids.

[28] Rivera H., Shibayama M., Tsutsumi V., Perez-Alvarez V., Muriel P. (2008). Resveratrol and trimethylated resveratrol protect from acute liver damage induced by CCl_4_ in the rat. J. Appl. Toxicol..

[29] Seif El-Din S.H., El-Lakkany N.M., Salem M.B., Hammam O.A., Saleh S., Botros S.S. (2016). Resveratrol mitigates hepatic injury in rats by regulating oxidative stress, nuclear factor-kappa B, and apoptosis. J. Adv. Pharm. Technol. Res..

[30] Uchida K. (2003). 4-Hydroxy-2-nonenal: A product and mediator of oxidative stress. Prog. Lipid Res..

[31] Shi C., Li Y., You Z., Tian Y., Zhu X., Xu H., Yang M., Zhang Y., Dong R., Quan H. (2024). Mangiferin Ameliorates CCl_4_-Triggered Acute Liver Injury by Inhibiting Inflammatory Response and Oxidative Stress: Involving the Nrf2-ARE Pathway. J. Inflamm. Res..

[32] Saha S., Buttari B., Panieri E., Profumo E., Saso L. (2020). An Overview of Nrf2 Signaling Pathway and Its Role in Inflammation. Molecules.

[33] Li L., Lan Y., Wang F., Gao T. (2023). Linarin Protects Against CCl_4_-Induced Acute Liver Injury via Activating Autophagy and Inhibiting the Inflammatory Response: Involving the TLR4/MAPK/Nrf2 Pathway. Drug Des. Dev. Ther..

[34] Lyu H., Wang H., Li L., Zhu J., Chen F., Chen Y., Liu C., Fu J., Yang B., Zhang Q. (2020). Hepatocyte-specific deficiency of *Nrf2* exacerbates carbon tetrachloride-induced liver fibrosis via aggravated hepatocyte injury and subsequent inflammatory and fibrogenic responses. Free Radic. Biol. Med..

[35] Xu W., Hellerbrand C., Kohler U.A., Bugnon P., Kan Y.W., Werner S., Beyer T.A. (2008). The Nrf2 transcription factor protects from toxin-induced liver injury and fibrosis. Lab. Investig..

[36] Wang B., Cui S., Mao B., Zhang Q., Tian F., Zhao J., Tang X., Chen W. (2022). Cyanidin Alleviated CCl_4_-Induced Acute Liver Injury by Regulating the Nrf2 and NF-κB Signaling Pathways. Antioxidants.

[37] Farkhondeh T., Folgado S.L., Pourbagher-Shahri A.M., Ashrafizadeh M., Samarghandian S. (2020). The therapeutic effect of resveratrol: Focusing on the Nrf2 signaling pathway. Biomed. Pharmacother..

[38] Kiso K., Ueno S., Fukuda M., Ichi I., Kobayashi K., Sakai T., Fukui K., Kojo S. (2012). The Role of Kupffer Cells in Carbon Tetrachloride Intoxication in Mice. Biol. Pharm. Bull..

[39] Brempelis K.J., Crispe I.N. (2016). Infiltrating monocytes in liver injury and repair. Clin. Transl. Immunol..

[40] Su L., Li N., Tang H., Lou Z., Chong X., Zhang C., Su J., Dong X. (2018). Kupffer cell-derived TNF-α promotes hepatocytes to produce CXCL1 and mobilize neutrophils in response to necrotic cells. Cell Death Dis..

[41] Tian Y., Ma J., Wang W., Zhang L., Xu J., Wang K., Li D. (2016). Resveratrol supplement inhibited the NF-κB inflammation pathway through activating AMPKα-SIRT1 pathway in mice with fatty liver. Mol. Cell. Biochem..

