# Supplementary material for: Protective Effects of Vitis coignetiae Vine Stem Extract Against Carbon Tetrachloride-Induced Acute Liver Injury in Mice
Source: Antioxidants (Basel). 2026 May 21;15(5):651. doi: 10.3390/antiox15050651 (PMC13203357; doi:10.3390/antiox15050651)
Supplement: Supplementary file 1 [file antioxidants-15-00651-s001.zip › antioxidants-4296225-supplementary.pdf]

*Supplementary Information*

# Protective Effects of *Vitis coignetiae* Vine Stem Extract against Carbon Tetrachloride-induced Acute Liver Injury in Mice

**Nam-Kyu Yoon [1], Jeongjun Lee [2], Hunsuk Chung [2], Jae-Kwang Kim [3,*], and Sae-Kwang Ku [1,*]**

[1] Department of Anatomy and Histology, College of Korean Medicine, Daegu Haany University, Gyeongsan 38610, Republic of Korea; yoonnnamkyu@dhu.ac.kr (N.-K.Y.);
[2] GAPI BIO Co., Ltd., Hwaseong 18622, Republic of Korea; orglab@gapibio.co.kr (J.L.); hunsukchung@dongbang-chem.co.kr (H.C.)
[3] Department of Physiology, College of Korean Medicine, Daegu Haany University, Gyeongsan 38610, Republic of Korea
* Correspondence: kim-jk@dhu.ac.kr (J.-K.K.); gucci200@dhu.ac.kr (S.-K.K.)

Academic Editor: Miquel Mulero

Received: 17 April 2026
Revised: 12 May 2026
Accepted: 20 May 2026
Published: 21 May 2026

**Copyright:** © 2026 by the author. Licensee MDPI, Basel, Switzerland. This article is an open access article distributed under the terms and conditions of the Creative Commons Attribution (CC BY) license.

## 1. Determination of Resveratrol in Vine Stem Parts of *Vitis coignetiae* Pulliat ex Planch (Crimson Glory Vine; CG) Extract

High-performance liquid chromatography (HPLC) analysis was performed using an Agilent 1260 Infinity II system (Agilent, Wald-bronn, Germany) equipped with a UV detector at 320 nm. Separation was achieved on a CAPCELL PAK C18 UG120 column (4.6 × 250 mm, 5 µm). The mobile phase consisted of solvent A (water containing 0.05% trifluoroacetic acid, TFA) and solvent B (acetonitrile, ACN). Elution was carried out under gradient conditions as follows: 79% A and 21% B from 0 to 35 min, followed by a change to 5% A and 95% B at 36 min, which was maintained until 45 min. The flow rate was set at 1.0 mL/min, with an injection volume of 10 µL, and the column temperature was maintained at 30°C. Under these conditions, the resveratrol standard exhibited a retention time of approximately 21.9 min, and a corresponding peak was observed at a similar retention time in the CG sample (Fig. S1). Quantitative analysis based on peak area indicated that the resveratrol content in CGR was 1.12 mg/g.

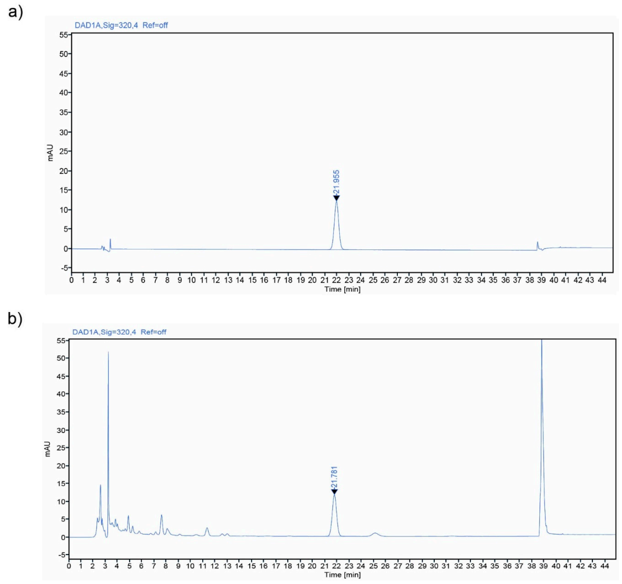


**Figure S1.** Identification of resveratrol in CG extract using high performance liquid chromatography (HPLC) analysis. (**a**) Chromatogram of resveratrol standard. (**b**) Chromatogram of CG.

https://doi.org/10.3390/antiox15050651
